# Single- vs. Multi-Walled Carbon Nanotubes: Differential Cellular Stress and Lipid Metabolism Effects in Macrophage Models

**DOI:** 10.3390/nano15181401

**Published:** 2025-09-11

**Authors:** Sara Nahle, Hilary Cassidy, David Matallanas, Bertrand H. Rihn, Olivier Joubert, Luc Ferrari

**Affiliations:** 1 Université de Lorraine, CNRS, IJL, F-54000 Nancy, France; sara-mkana@hotmail.com (S.N.); bertrand.rihn@univ-lorraine.fr (B.H.R.); luc.ferrari@univ-lorraine.fr (L.F.); 2 Systems Biology Ireland, School of Medicine, University College Dublin, Belfield, D04 V1W8 Dublin, Ireland; hilary.cassidy@ucd.ie (H.C.); david.gomez@ucd.ie (D.M.); 3 School of Biomolecular and Biomedical Sciences, University College Dublin, Belfield, D04 V1W8 Dublin, Ireland

**Keywords:** carbon nanotubes, omics, *in vitro*, macrophages, metabolism disruption, corona

## Abstract

This study examines the toxicological effects of carbon nanotubes (CNTs) of different diameters—single-walled CNTs (SWCNT, 2 nm) and multi-walled CNTs (MWCNT, 74 nm)—on two macrophage cell lines, rat alveolar NR8383 cells and human differentiated THP-1. Using standardized exposure conditions and employing an integrated omics approach (transcriptomic and proteomic analyses), both CNT types were found to induce cellular stress responses and inflammation, especially in NR8383 cells, with notable involvement of the Sirtuin signaling pathway. After 24 h, MWCNTs uniquely disrupted lipid metabolism in NR8383 cells, resulting in foam cell formation and syncytia. While SWCNTs were less disruptive to metabolic pathways, they significantly altered gene regulation, particularly RNA splicing mechanisms. The dispersion medium—fetal bovine serum (FBS) versus human surfactant—also modulated the observed toxicological responses, highlighting the critical role of the protein corona in influencing CNT-cell interactions. These findings demonstrate that CNT diameter significantly affects cytotoxicity and cellular response pathways in a cell-type-specific manner.

## 1. Introduction

In addition to their widespread applications in electronics, textiles, and energy storage, carbon nanotubes (CNTs) are emerging as promising materials in the biomedical field [1]. As drug carriers, CNTs show good promise in therapeutic applications [2,3], Including anticancer strategies, due to their ability to penetrate cells and localize within organelles such as mitochondria [4,5]. However, increasing levels of human exposure to CNTs have raised significant public health concerns, particularly in light of the lack of standardized safety protocols for their production and use. For instance, the NIOSH Current Intelligence Bulletin (CIB) has proposed an occupational exposure limit of 1 µg/m^3^ for CNTs, based on toxicological and animal data, while simultaneously emphasizing the need for further studies to fully characterize their potential health risks. In particular, the long-term effects of chronic exposure and the potential carcinogenicity of specific CNT types require comprehensive investigation.

Inflammatory responses following pulmonary administration of CNTs have been well documented in animal studies. Qin et al. demonstrated that the intravenous injection of CNT in rats resulted in persistent inflammation, granuloma formation, and pulmonary fibrosis [6]. Similarly, Shvedova et al. observed acute inflammation, granulomatous pneumonia, and oxidative stress following pharyngeal aspiration or inhalation of CNTs in C57BL/6 mice [7]. In line with these findings, Vietti identified CNT diameter and size as key factors influencing fibrotic potential [8]. Collectively, in vivo studies indicate that CNTs can trigger inflammatory responses and promote granuloma formation, ontributing to chronic respiratory diseases such as fibrosis, sarcoidosis, and lung cancer, depending on their physicochemical characteristics [9,10].

*In vitro* studies have supported previous findings by demonstrating CNT exposure induces oxidative stress, inflammatory reactions, and the expression of pro-fibrotic markers in various cell models, including lung epithelial cells [11] and human macrophages [12]. Although *in vitro* systems have inherent limitations in replicating the complexity of real-world exposure, they provide valuable insights into molecular mechanisms, key events, and toxicity pathways that underlie adverse outcomes observed *in vivo*. Considering that inhalation is the primary route of CNT exposure and that macrophages serve as the first line of immune defense against inhaled nanomaterials, rat alveolar macrophages (NR8383 cells) were selected for this study due to the absence of an established human alveolar macrophage cell line. In parallel, the human monocytic THP-1 cell line, differentiated into macrophages using phorbol 12-myristate 13-acetate (PMA)—referred to here as dTHP-1—was utilized to compare the responses between rodent and human macrophage models. Both cell types were exposed to CNTs of similar length (3–4 µm)s, pecifically SWCNT, and MWCNT.

Previous studies have demonstrated that both macrophage cell models exhibit increased oxidative stress and inflammation following exposure to CNT [13,14]. Interestingly, Lin et al. reported that MWCNTs specifically induced lipid accumulation in dTHP-1 cells, a phenomenon linked to endoplasmic reticulum stress [15]. This effect, however, has not been observed in NR8383 cells. Comparative analysis of these two cell lines under standardized conditions enables the identification of both shared and cell-type-specific responses to CNT exposure.

In this contect,. the protein coronas formed on CNTs and the proteomes of exposed dTHP-1 cells were analyzed. The hypophase surfactant (HS), representing the first biological barrier encountered by inhaled nanomaterials in the alveolar space, is known to interact with nanomaterials and alter their biological identity [16]. Several studies have evaluated the role of HS in modifying nanomaterial toxicity [17,18], primarily through its influence on cellular uptake and clearance mechanisms. For example, HS has been shown to reduce the toxicity of silver nanoparticles in human alveolar type-I-like epithelial cells [19] while increasing the cytotoxicity of silica nanoparticles in an *in vitro* air-blood barrier model [20]. Based on this evidence, the present study investigates whether CNT coating by HS components alters their toxicological profile.

The present study aims to elucidate the comparative toxicity of two CNTs (a MWCNT (NRCWE-006, better known as Mitsui-7) and a sSWCNT designated NRCWE-055)—which differ in diameter but share similar lengths. A dual cell line approach was employed to investigate the underlying molecular mechanisms associated with CNT exposure. In addition, this study examined the influence of the protein corona within these *in vitro* models to identify key events potentially that may be predictive of the in vivo responses. Although *in vitro* studies cannot fully replicate the complexity of real-world exposure scenarios, they offer valuable insights into the underlying molecular mechanisms, critical events, and toxicity pathways associated with observed adverse effects in vivo. Understanding these mechanistic pathways is essential for predicting in vivo responses, particularly in light of existing evidence demonstrating that CNT exposure can induce inflammation, potential granuloma formation, and lipid metabolism disruption, highlighting a significant public health risk.

## 2. Materials and Methods

### 2.1. Carbon Nanotubes

Multi-walled carbon nanotubes (MWCNT) designated NRCWE-006 (Mitsui-7) and single-walled carbon nanotubes (SWCNT) designated NRCWE-055 were procured from the National Research Centre for the Working Environment (NRCWE) located in Copenhagen, Denmark. The supplier provided information on the length, diameter, metal impurities, slope fluorescein, and specific surface area of the CNT, as determined by the Brunauer–Emmett–Teller (BET) method (Table 1). Stock solutions of CNTs were prepared in a clean area using dry and clean nanopowders.

### 2.2. Characterization of CNT

Dry nanopowders were suspended at 2 mg/mL in Dulbecco’s Modified Eagle Medium (DMEM, high glucose) supplemented with 2% fetal bovine serum (FBS; Sigma–Aldrich, St. Louis, MO, USA). For comparative corona analysis, an alternative dispersion solution of DMEM high glucose with 1% human surfactant (HS) and 1% human albumin (HA; Sigma–Aldrich) was employed. Carbon nanotubes (CNTs) were then sonicated using a Vibra Cell™ Sonicator (VWR, Lutterworth, UK) for 15 min at 10% amplitude for NRCWE-055 and 30% amplitude for Mitsui-7. Following sonication, CNT suspensions were diluted to the desired working concentrations in DMEM without FBS or HS/HA.

Dynamic Light Scattering (DLS): The hydrodynamic size of each CNT was measured using a ZetaSizer™ (Malvern Inc., Malvern, Worcs, UK) immediately after suspension in cell media (with FBS or HA + HS) (Table 1).

**Figure 1:**
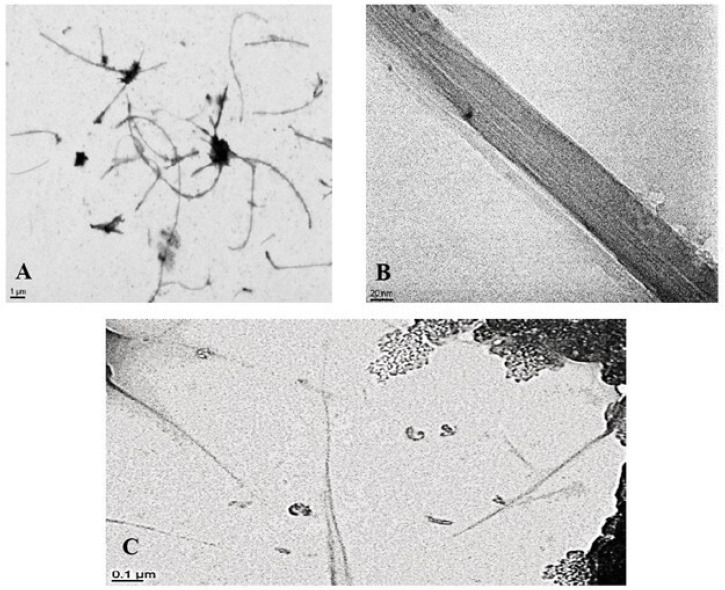
TEM images of (**A**,**B**) MWCNT and (**C**) SWCNT dispersed in DMEM high glucose with 2% FBS.

Transmission Electron Microscopy (TEM): The CNT shape was characterized by TEM. A drop of each CNT suspension was placed on a carbon-coated copper grid and air-dried before observation using an ARM 200F microscope (Philips, Amsterdam, The Netherlands) operated at 200 kV (Figure 1).

### 2.3. Cell Culture

NR8383 rat alveolar macrophages (ATCC^®^ CRL-2192™) and THP-1 human monocytic leukemia cells (ATCC^®^ TIB-202) were obtained from the American Type Culture Collection (Manassas, VA, USA). Both cell lines were cultured in DMEM (Dulbecco’s Modified Eagle’s Medium, high glucose) supplemented with 15% heat-inactivated FBS, 100 U/mL penicillin, 100 µg/mL streptomycin, 4 mM L-glutamine, and 0.25 µg/mL amphotericin B (Sigma-Aldrich, St. Louis, MO, USA). Cells were incubated at 37 °C in a humidified atmosphere with 5% CO_2_. For all experiments, cells were seeded 24 h prior to CNT exposure at a density of 5 × 10^4^ cells/mL. THP-1 monocytes were differentiated into macrophages dTHP-1 by treatment with 10 ng/mL phorbol 12-myristate 13-acetate (PMA) for 24 h as previously described [21].

### 2.4. Cytotoxicity and Mitochondrial Activity Assays

Lactate dehydrogenase (LDH) leakage was assessed using the LDH assay kit (Roche, Boulogne, France) following the manufacturer’s instructions. NR8383 and dTHP-1 cells were seeded in 96-well plates and exposed to varying CNT concentrations (0–300 cm^2^/cm^2^ for SWCNT and 0–16 cm^2^/cm^2^ for MWCNT, equivalent to 0–200 µg/mL). dTHP-1 cells were exposed to CNTs dispersed in both 2% FBS (as with NR8383 cells) and 1% HS + 1% HA to evaluate the influence of dispersion media on cytotoxicity. After 24 h, plates were centrifuged (800× *g*, 10 min), and 100 µL of each supernatant was transferred to a new 96-well plate containing 100 µL of LDH reaction mixture. Following a 30-min incubation at room temperature, 50 µL of stop solution was added, and absorbance was measured at 490 nm using an iMark™ Microplate Reader (Bio-Rad Laboratories, Osaka, Japan). Untreated cells (negative control) and cells treated with 10% Triton X-100 (positive control) were included.

Mitochondrial dehydrogenase activity was assessed using the WST-1 Cell Proliferation Reagent (Roche, Boulogne, France) according to the manufacturer’s protocol. After 24 h of CNT exposure, WST-1 reagent was added to each well, and cells were incubated at 37 °C for 2 h. Absorbance was measured at 450 nm using the iMark™ Microplate Reader. The inhibitory concentration (IC50) was calculated using the Reed–Muench method [22]. Untreated cells (negative control) and cells treated with 5 µL DMSO (positive control) were included. Cell viability data are presented as means  ±  standard error of the mean (SE) of four biological replicates. Statistical differences were determined by one-way analysis of variance (ANOVA) followed by Dunnett’s test using GraphPad Prism 8 software™.

#### Dose Selection and Experimental Replicates

Following the approach described by Schmid and Cassee [23], we expressed the exposure dose as the surface area of CNTs relative to the surface area of cells (cm^2^/cm^2^), in order to better reflect the interaction potential between nanomaterials and the cell monolayer. The CNT surface areas were provided by the manufacturer (see Table 1).

To determine appropriate subtoxic concentrations for downstream analyses, we performed WST-1 cytotoxicity assays after 4 h and 24 h of exposure to calculate IC_50_ values. A quarter of the IC_50_ (¼ IC_50_) was then used as a working concentration. This approach allowed us to avoid significant cell death while inducing an early stress response, which is particularly relevant for studying initial signaling events. For transcriptomic analysis, a 4 h exposure at ¼ IC_50_ was chosen to capture the early phase of gene expression changes. For proteomic analysis, the same concentration was applied for 24 h in order to assess which of these early responses were translated and maintained at the protein level. This design allows comparison between early transcriptional signals and later sustained protein-level responses. In the case of MWCNTs, cytotoxicity was very low, and the IC_50_ exceeded the highest tested concentration. Therefore, we selected the lowest concentration that showed measurable cytotoxic effects and applied this same dose for both NR-883 and THP-1 cells to maintain consistency across cell models.

All experiments were performed using four independent biological replicates. For cytotoxicity assays, six technical replicates were used per condition, while four technical replicates were included for both transcriptomic and proteomic analyses.

### 2.5. RNA Analysis and Microarrays

#### 2.5.1. RNA Isolation and Quantification

Total RNA was extracted from NR8383 and dTHP-1 cells exposed for 4 h to MWCNT (1 cm^2^/cm^2^) and SWCNT (11 cm^2^/cm^2^) dispersed in FBS using Trizol Reagent (Omega Bio-Tek, Guangzhou, China). These doses, selected based on NR8383 WST-1 results (as dTHP-1 IC50s were higher than the tested doses), were also used for dTHP-1 exposures for direct comparative purposes. The rationale for these dose selections is provided in the Section 3. Untreated cells served as controls. Following cell lysis, chloroform (Carlo Erba Reagents, Normandie, France) was added, and samples were centrifuged (10,000× *g*, 5 min). The supernatant was mixed with isopropanol (Carlo Erba Reagents), and the resulting precipitate was washed with 80% ethanol, incubated at −20 °C, and dissolved in RNase-free water. RNA concentration was determined at 260 nm using a BioSpec-nano Spectrophotometer (Shimadzu, Kyoto, Japan). RNA purity was confirmed by an A260/A280 ratio > 1.8. RNA integrity was assessed using the RNA 6000 Nano Reagents Kit and Bioanalyzer 2100 (Agilent Technologies, Waldbronn, Germany), with a RIN cutoff score of 8.

#### 2.5.2. Microarray Expression Profiling

Microarray sample preparation was performed as previously described [24]. cRNA synthesis, Cy3-dye labeling, hybridization, and washing were conducted using 100 ng of total RNA and Agilent Low Input Quick Amp Labeling kits (Agilent Technologies). Microarray slides (Agilent Technologies) were scanned using an Agilent G2505C DNA microarray scanner with specified settings (one-color channel for 8 × 60 k arrays; 61 × 21.6 mm scan area; 3 µm resolution; Green dye channel; 20-bit TIFF files; Green PMT at 100%). TIFF images and fluorescence signal quantification were acquired using Agilent Feature Extraction software version 11.0.1.1.

#### 2.5.3. Transcriptomic Data Analysis

Quality control-validated data were normalized using GeneSpring GX 13.0 software (Silicon Genetics, Redwood City, CA, USA). Differentially expressed genes were identified using a *p*-value < 0.001 and a fold change > |1.5|, with statistical analysis performed using Benjamini–Hochberg False Discovery Rate correction. Gene ontology and pathway analysis were performed using Ingenuity Pathway Analysis (IPA, Qiagen Bioinformatics, Redwood City, CA, USA), DAVID Functional Annotation Bioinformatics Microarray Analysis (https://david.ncifcrf.gov/, accessed on 23 February 2022), and STRING (https://string-db.org/ accessed on 22 March 2022) for protein association network analysis.

### 2.6. Protein Analysis

#### 2.6.1. Sample Preparation for Proteomics

Single-pot solid-phase-enhanced sample preparation (SP3) using carboxylate-modified magnetic beads (GE Healthcare, Chicago, IL, USA) was employed to analyze the global proteome of NR8383 and dTHP-1 cells exposed to CNTs dispersed in FBS (at the same doses used for transcriptomics). Additionally, the dTHP-1 proteome was assessed for CNTs dispersed in 1% HS + 1% HA. Cells were exposed for 24 h to MWCNT (1 cm^2^/cm^2^) and SWCNT (11 cm^2^/cm^2^). Lysis was performed in a buffer containing 6 M urea, 2 M thiourea, and 50 mM MOPS. Samples were reduced with DTT and alkylated with IAA. A 1:1 mixture of hydrophobic and hydrophilic Sera-Mag SpeedBead carboxylate-modified magnetic particles was added to each sample. Following immobilization and washing, proteins and peptides were eluted with MS-grade water (Fisher Scientific, Oslo, Norway).

Supernatants from NR8383 and dTHP-1 cell exposures (as described above) were also processed. Protein content was determined using a NanoDrop 2000 spectrophotometer (Thermo Scientific, Waltham, MA, USA). Filter-aided sample preparation (FASP) was performed; 50 µg of protein was reduced with DTT, mixed with 8 M urea in 0.1 M Tris-HCl, alkylated with iodoacetamide, and digested overnight at 37 °C with sequence-grade trypsin (Promega, Madison, WI, USA) at a 1:50 (*w*/*w*) enzyme-to-protein ratio. Peptides were recovered by centrifugation, washed with 50 mM NH_4_HCO_3_, and centrifuged again. Protein concentrations were measured using a NanoDrop 2000; 20 µg of tryptic digests were desalted on C18 Stage tips. Following elution and lyophilization, samples were resuspended in TFA.

#### 2.6.2. Mass Spectrometry

Each treatment, with four biological replicates, was analyzed on a Thermo Scientific Q Exactive mass spectrometer coupled to a Dionex Ultimate 3000 RSLCnano chromatography system (hermo Fisher Scientific, Waltham, MA, USA). Samples were loaded onto a fused silica emitter (75 µm ID) packed with Reprocil Pur C18 (1.9 µm, 12 cm) reverse phase media (Dr. Maisch High Performance LC GmbH, Ammerbuch-Entringen, Baden-Württemberg, Germany). Peptides were separated using a 90-min acetonitrile gradient at a 250 nL/min flow rate. The MS was operated in positive ion mode (320 °C capillary temperature, 2300 V frit potential) using data-dependent acquisition. A high-resolution (70,000) MS scan (300–1600 *m*/*z*) was performed, and the 12 most intense ions were selected for MS/MS analysis using high-energy collision dissociation (HCD).

#### 2.6.3. Data Analysis

Proteins were identified and quantified by MaxLFQ using MaxQuant version 1.5 against the Homo sapiens reference proteome database (Uniprot). Carbamylation (C) and oxidation (M) were set as fixed and variable modifications, respectively. Data were analyzed using IPA and STRING platforms.

### 2.7. May-Grünwald Giemsa (MGG) Staining

NR8383 cells were cultured at a density of 1 million cells per 20 mL in DMEM with 2% FBS at 37 °C and 5% CO_2_. After 24 h, cells were scraped, collected, and centrifuged (130× *g*, 5 min). The following day, cells were exposed to MWCNT (1 cm^2^/cm^2^) and SWCNT (11 cm^2^/cm^2^) for 4, 24 h, and 3 days, with three replicates per condition. Untreated controls were included for each time point. Following exposure, cells were collected, centrifuged (130× *g*, 5 min), and resuspended in 500 µL PBS. Cell suspensions were then smeared onto slides and allowed to air dry for at least 30 min. Dried slides were stained using the RAL 555 Kit (RAL Diagnostics, Martillac, France) according to the manufacturer’s instructions, as follows: 5 s in fixative, 5 s in eosin, and 3 s in methylene azure. Slides were then observed under an optical microscope at 400× magnification. Data were shown in the Figure S5.

## 3. Results

### 3.1. Characterization of CNT

The length and structure of carbon nanotubes (CNTs) were characterized using transmission electron microscopy (TEM). Figure 1 presents representative TEM images of Mitsui-7 (multi-walled carbon nanotubes, MWCNTs) and NRCWE-055 (single-walled carbon nanotubes, SWCNTs) dispersed in media containing 2% fetal bovine serum (FBS) after 15 min of sonication. The approximate lengths of both MWCNTs and SWCNTs were comparable, measuring approximately 4 µm and 3 µm, respectively. However, the MWCNTs exhibited a larger diameter (65 nm) compared to the SWCNTs (12 nm) (Table 1). The percentage of impurities present in each CNT type is summarized in Table 1. SWCNTs demonstrated a higher overall metal impurity content (1.4%) compared to MWCNTs (0.0004%), particularly with respect to iron and cobalt. Furthermore, SWCNTs exhibited a greater capacity for reactive oxygen species (ROS) generation, as indicated by a higher fluorescein slope compared to MWCNTs (Table 1).

### 3.2. Cytotoxic Effects of Carbon Nanotubes on NR8383 and dTHP-1 Cells

#### 3.2.1. Cell Viability of Alveolar Macrophages (NR8383)

**Figure 2:**
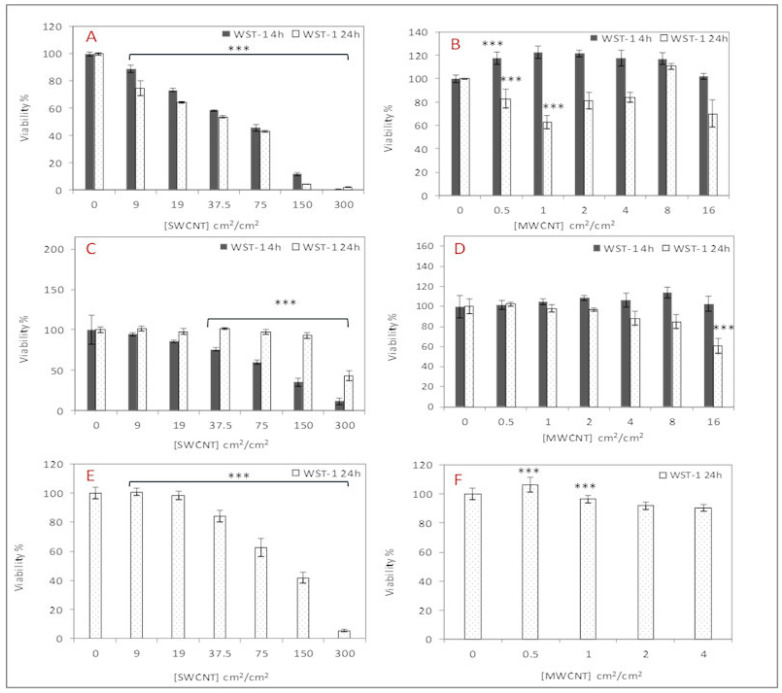
Mean Dose-Dependent Cytotoxicity of SWCNTs and MWCNTs in NR8383 and dTHP-1 Cells. (**A**) NR8383 cells exposed to SWCNTs in FBS; (**B**) NR8383 cells exposed to MWCNTs in FBS; (**C**) dTHP-1 cells exposed to SWCNTs in FBS; (**D**) dTHP-1 cells exposed to MWCNTs in FBS; (**E**) dTHP-1 cells exposed to SWCNTs in HS + HA; (**F**) dTHP-1 cells exposed to MWCNTs in HS + HA. n = 6, N = 4, *** *p* < 0.001.

Exposure of rat alveolar macrophages (NR8383) to SWCNTs resulted in a significant, concentration- and time-dependent decrease in cell viability (Figure 2). The 24-h IC50 for SWCNTs was determined to be 44 cm^2^/cm^2^. A subtoxic concentration of ¼ IC50 (11 cm^2^/cm^2^) was selected for subsequent transcriptomic and proteomic analyses to investigate the primary cellular response to SWCNT exposure. In contrast, MWCNT exposure did not elicit a dose-dependent cytotoxic effect at either 4 or 24 h. The maximum reduction in cell viability observed with MWCNTs was approximately 40% at 1 cm^2^/cm^2^ after 24 h. Notably, a significant increase in cell viability was observed at 0.5 and 1 cm^2^/cm^2^ at the 4-h time point. The 24-h IC50 for MWCNTs exceeded the highest tested dose. Therefore, 1 cm^2^/cm^2^ was chosen for further experiments due to the observed biological response at this concentration. In summary, SWCNTs exhibited greater cytotoxicity towards NR8383 cells compared to MWCNTs across the tested concentrations. 

#### 3.2.2. Cell Viability of dTHP-1 Cells

SWCNTs dispersed in fetal bovine serum (FBS) induced minimal cytotoxicity in dTHP-1 cells, with only approximately 60% cell viability observed at the highest concentration (300 cm^2^/cm^2^). This indicates that SWCNTs dispersed in FBS are less cytotoxic to dTHP-1 cells compared to NR8383 cells (Figure 2). The dispersion of SWCNTs in human surfactant (HS) and hyaluronic acid (HA) resulted in increased cytotoxicity in dTHP-1 cells, with an IC50 of 104 cm^2^/cm^2^. MWCNTs dispersed in FBS did not induce significant cytotoxicity in dTHP-1 cells; only approximately 40% cell reduction was observed at the highest concentration (16 cm^2^/cm^2^). No significant differences in cytotoxicity were observed in dTHP-1 cells between MWCNTs dispersed in HS and HA compared to those dispersed in FBS, except at the highest concentration, where HS and HA dispersion resulted in a significant decrease in cytotoxicity

### 3.3. Genome Modulation

To investigate the impact of carbon nanotube (CNT) exposure on rat (NR8383) and human (dTHP-1) macrophage genomes, transcriptomic analyses were performed, focusing on primary responses to sub-toxic CNT doses. Two CNTs with similar length ranges but differing diameters were compared: a single-walled CNT (SWCNT, NRCWE-055) and the multi-walled CNT (MWCNT, Mitsui-7).

**Figure 3:**
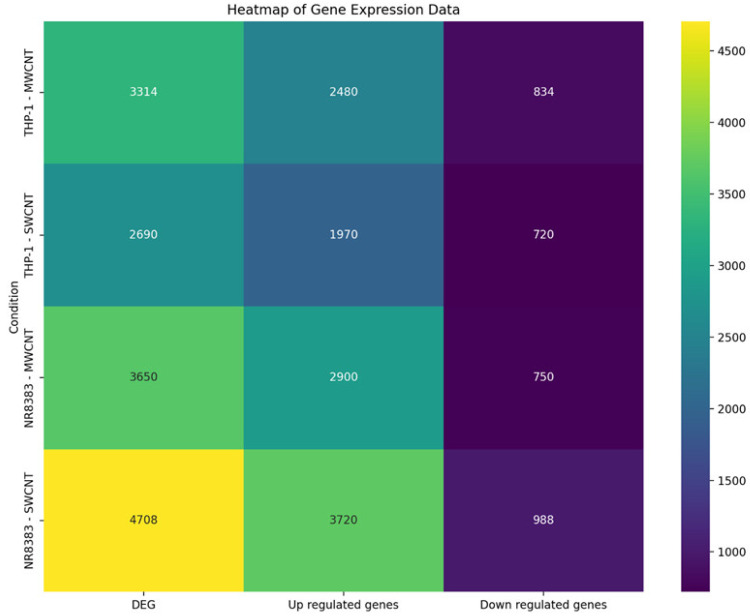
Gene expression profiles in macrophages exposed to carbon nanotubes: a heatmap visualization.

CNT exposure induced significant gene expression alterations in both cell types. NR8383 cells exhibited greater gene expression variation than dTHP-1 cells for both CNTs, correlating with cytotoxicity data. SWCNT exposure resulted in a higher number of differentially expressed genes (DEGs) in both cell types. Upregulation was observed for 80% of DEGs in dTHP-1 cells and 73–74% in NR8383 cells, irrespective of CNT type. The analysis of shared transcriptomic changes revealed 431 common DEGs across both cell types and CNTs, alongside unique DEGs for each exposure: 241 (SWCNT) and 583 (MWCNT) in dTHP-1 cells, and 875 (SWCNT) and 645 (MWCNT) in NR8383 cells (Figure 3).

**Figure 4:**
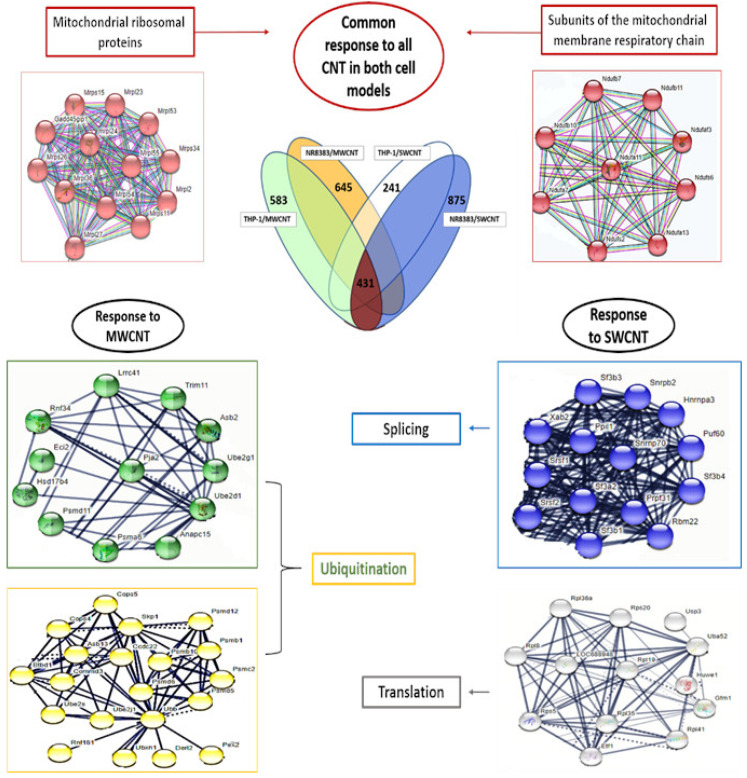
Transcriptomic Response of NR8383 and dTHP-1 Macrophages to 4-h Exposure to SWCNTs and MWCNTs: Venn diagram and protein-protein interaction analysis (String, *p*-value = 0.001, FC > 1.5, high confidence).

Protein-protein interaction analysis (String, *p*-value = 0.001, FC > 1.5, high confidence) of these gene groups (Figure 4) identified common gene clusters associated with mitochondrial dysfunction, including mitochondrial ribosomal proteins and ubiquinone oxidoreductase subunits. Exposure-specific DEG clusters revealed enrichment for splicing activity (SWCNT, NR8383), translation activity (SWCNT, dTHP-1), and post-translational protein modifications, particularly ubiquitination (MWCNT, both cell types).

**Figure 5:**
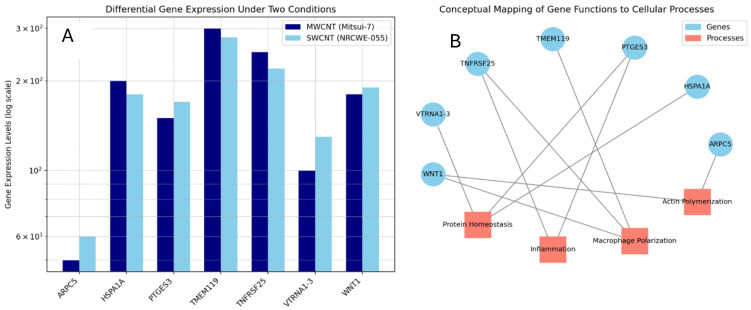
Fold-change values of the most significantly upregulated genes common to dTHP-1 cells exposed to MWCNTs and SWCNTs (**A**), and their associated molecular functions (**B**).

Among the top ten upregulated DEGs (*p*-value < 0.001, FC > 1.5) (Table S1) for each exposure, seven were shared between both CNTs in dTHP-1 cells (Figure 5). These genes were associated with inflammatory response (Cxcl2, Ccl2, Tnfrsf25) and macrophage polarization (Tac4, Osm, Gdf15, Phf19, Wnt1, Csrnp1), particularly in NR8383 cells exposed to SWCNT as well as in actin polymerization.

A transcriptomic proliferation signature was observed with both CNTs in both cell types, including shared DEGs such as TIAM1, RASA1, and PDGFRB (dTHP-1) and Tp53INP1, Igf1, Mki67, and Rictor (NR8383).

**Figure 6:**
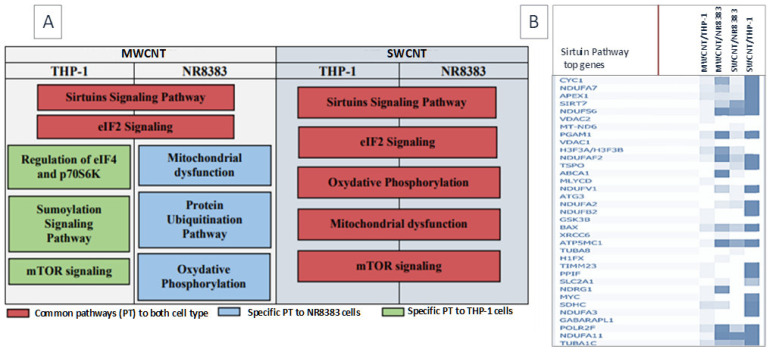
(**A**) Top five canonical pathways dysregulated in NR8383 and dTHP-1 cells after MWCNT and SWCNT exposure; (**B**) expression levels of key Sirtuin signaling pathway genes across all exposures.

Pathway analysis (IPA, *p*-value = 0.001, FC > 1.5) identified “Sirtuin signaling pathway” and “Eif2 signaling pathway” as commonly dysregulated pathways (Figure 6), with the former exhibiting greater dysregulation in NR8383 cells, particularly with SWCNT exposure (Overlay: 38.7% for SWCNT > 25.9% for MWCNT, *p*-value: 4.16 × 10^−24^ for SWCNT < 5.97 × 10^−11^ for MWCNT). Mitochondrial perturbation, identified via String analysis, was confirmed by IPA, with SWCNT inducing mitochondrial dysfunction pathway deregulation in both cell types, and MWCNT affecting this pathway in NR8383 cells only, suggesting mitochondria as a common CNT target. The “Oxidative phosphorylation pathway” deregulation is accompanied by “Mitochondrial dysfunction pathway” deregulation.

### 3.4. Proteome Modulation

**Figure 7:**
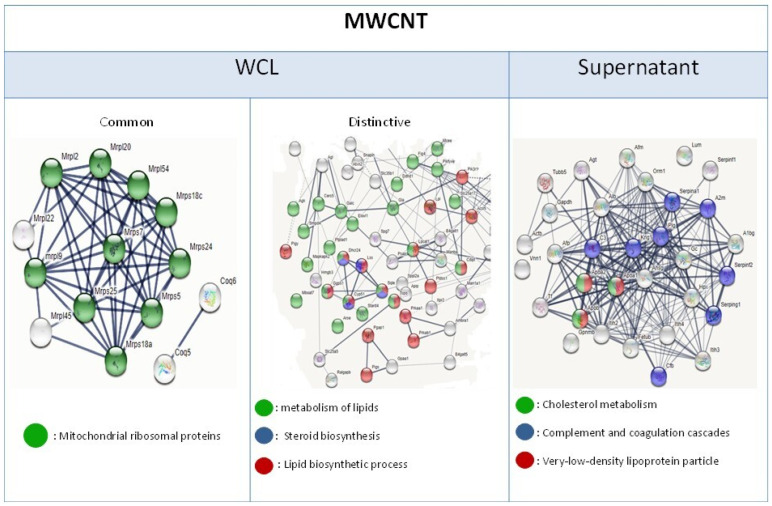
Common and Distinct Differentially Expressed Proteins in NR8383 Cells After 24-h MWCNT Exposure: WCL and Supernatant. White circles are proteins all from different pathways.

To determine the translational impact of gene expression variations and elucidate the persistent stress responses induced by carbon nanotubes (CNTs) in macrophages, a comparative transcriptomic and proteomic analysis was conducted. We focused on identifying common gene and protein expression patterns. NR8383 cells exposed to multi-walled CNTs (MWCNTs) exhibited 142 differentially expressed proteins (DEPs), also identified as differentially expressed genes (DEGs) in whole cell lysates (WCLs). A significant cluster of these common DEPs was associated with mitochondrial ribosomal proteins. DEPs identified only at the protein level were enriched in lipid metabolism pathways. Supernatant DEPs were also related to lipid metabolism and complement/coagulation cascades (Figure 7).

**Figure 8:**
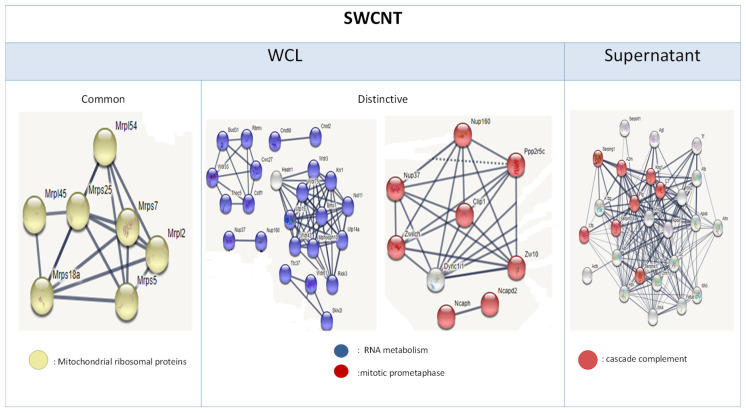
Common and Distinct Differentially Expressed Proteins in NR8383 Cells After 24-h SWCNT Exposure: WCL and Supernatant.

Following single-walled CNT (SWCNT) exposure in NR8383 cells, 190 common DEPs were identified, including a cluster of mitochondrial ribosomal proteins. Protein-level specific DEPs were associated with RNA metabolism and mitotic prometaphase. Consistent with MWCNT exposure, supernatant DEPs were linked to complement and coagulation cascades (Figure 8).

In differentiated dTHP-1 cells exposed to MWCNTs, only 8 DEGs were identified among 515 DEPs (DNPEP, EIF4G3, EXOC5, GSTM4, MAGT1, NCEH1, RAB7B, SELENOT). Notably, 50 DEPs were related to lipid metabolism, and mitochondrial ribosomal proteins were also identified (Figure S1). 

Supernatants from both CNT exposures in dTHP-1 cells contained 93 common DEPs among 124 (MWCNT) and 133 (SWCNT), primarily associated with regulated exocytosis and innate immune response (Figure S2).

Both CNTs, dispersed in fetal bovine serum (FBS) or low serum (LS) media, induced proteins implicated in immune response, vesicle-mediated transport, and regulated exocytosis. dTHP-1 cell WCLs and supernatants exposed to CNTs dispersed in FBS exhibited a higher number of DEPs compared to those dispersed in LS, particularly for SWCNTs. WCLs from FBS-dispersed CNT exposures showed DEPs related to purine ribonucleotide binding, while LS-dispersed exposures revealed DEPs associated with syntaxin and SNARE binding (MWCNT) or mitochondrial binding (SWCNT). Supernatants from LS-dispersed CNT exposures showed DEPs related to responses to toxic substances with both CNTs (Figures S3 and S4).

## 4. Discussion

In the present work, we have performed a genome and proteome-wide study on the effect of two types of CNT, SWCNT and MWCNT from a similar length range, on two macrophage models, NR8383 and dTHP-1 differentiated with PMA. After assessing the cytotoxicity study, we obtained higher cell reduction with SWCNT than MWCNT in both cell types. This result was also found by Jia et al., SWCNT induces higher cytotoxicity in alveolar macrophages than MWCNT [25]. Kumarathasan et al. showed a positive correlation between surface area, metal content, and cellular ATP, which can explain our result; SWCNT, which has higher surface area and metal content compared to MWCNT, undergoes higher mitochondrial damage according to the WST-1 test [26]. Also, Knirsh et al. show that metal content increases SWCNT toxicity in NR8383 cells using the WST-1 test [27]. This higher cytotoxicity of this SWCNT can also be due to its higher oxidative potential, as it was concluded by Kim et al. [28]. Cytotoxicity increased when we dispersed these SWCNT with surfactant compared to those dispersed in FBS, especially at higher doses. It may be due to the high dispersion of SWCNT in HS and albumin [29]. The cytotoxicity endpoints were steeper with NR8383 cells compared to dTHP-1 cells with both CNTs. Both cell lines, besides originating from different species, have different phenotypes, as dTHP-1 differentiated with PMA are adherent cells, whereas NR8383, a monocyte-macrophage cell line [30], exhibits 50% adherent and 50% non-adherent cells. Monocytes have shown a higher uptake of monocrystalline iron oxide (MION) particles than peritoneal macrophages, which is attributed to the variant differentiation stage of the phagocytes [31]. Thus, we suggest that NR8383 cells were more damaged by these CNTs because CNT uptake was higher in NR8383 cells than in dTHP-1 cells. Moreover, gene expression was also altered more in NR8383 cells than in dTHP-1 cells with both CNT, which is consistent with cytotoxicity results.

Both CNTs damage the mitochondria in both cell models; interestingly, they alter mitochondrial ribosomal proteins (MRP) expression, which was not seen, to our knowledge, with CNT in previous studies. Mitoribosomes activity disruption leads to oxidative phosphorylation impairment, thus altering macrophage growth and differentiation [32]. So, it was obvious that MWCNT/Mitsui-7, having the lowest cytotoxic effect on dTHP-1 cells compared to its effect on NR8383 cells and to SWCNT exposures in both cell models, does not significantly disrupt mitochondrial and oxidative phosphorylation pathways in this cell model (Figure 6). Another common response to CNT in both cell types was the dysregulation of the Sirtuin signaling pathway. Some Sirtuin-like SIRT3 are localized exclusively in mitochondria, and SIRT3 can regulate characteristic mitochondrial processes like protein deacetylation. The perturbation of these actors leads to mitochondrial dysfunctions, inflammation, oxidative stress, and lipid accumulation. Therefore, we think that CNT, by targeting the mitochondria, stimulates Sirtuin signaling. To our knowledge, CNT exposure was never correlated with Sirtuin pathway deregulation, although these histone deacetylases may be an interesting regulator of CNT effects in macrophages, especially since they are implicated in some key events and adverse outcomes associated with CNT exposure, like oxidative stress, acute and chronic inflammation, fibrosis, and cancer. Testing only MWCNTs, Yang et al. also observed that MWCNTs induce lipid accumulation [33]. While they used shorter CNTs (<2 µm), our results demonstrate that both long MWCNTs and long NWCNTs (length > 2 µm) similarly promote lipid accumulation. This reinforces the notion that lipid accumulation is a general response to CNT exposure, independent of CNT length within the tested range. In NR8383 cells, MWCNT stimulates ubiquitin proteasome activity, resulting in an overexpression of Psmc2, Psmd6, and Ube2, among others (Figure 8). This activation would be in response to reticulum endoplasmic stress (RES) as ubiquitination is required for the efficient removal and degradation of misfolded proteins [34]. The dysregulation of mTOR, eif2, and Sirtuin signaling pathways, in addition to the overexpression of several ribosomal proteins Rps14, Rps27, and Xbp-1, confirms the induction of RES after NR8383 exposure to MWCNT [35]. This result was found by Zhao et al., who show that MWCNT induced RES in HUVECs cells [36] and in dTHP-1 cells [37]. In their first study, they prove that RES was MWCNT diameter dependent, and in their second study, they associate that stress with lipid accumulation. This finding is consistent with our results. As we can see in Figure S5, we observed foamy macrophages after NR8383 exposure to MWCNT for 4 h and 24 h, using MGG staining.

At the gene expression level, downregulation of the Abca1 gene—known to promote foam cell formation—was observed [38]. Whole cell lysate (WCL) proteome analysis of NR8383 cells exposed to MWCNTs revealed perturbations in lipid metabolism (Figure 7), and deregulation of the cholesterol biosynthesis pathway (Figure S1). Apolipoproteins Apoa1 and Apoa2 were detected in bothWCL and supernatants, suggesting that alveolar macrophages may upregulate apolipoproteins to enhance cholesterol efflux from foam cell macrophages and thereby reduce lipid accumulation [39]. These findings indicate that MWCNTs contribute to lipid metabolism disruption and form cell macrophage formation potentially through impairment of endoplasmic reticulum function. In addition, deregulation of the sirtuin pathway—also implicated in lipid metabolism—may represent either a contributing factor or a compensatory mechanism in response to this metabolic stress [40,41] Notably, Sirt3, which has been shown to inhibit lipid accumulation in macrophages, was overexpressed in NR8383 cells after 4 h MWCNT exposure, further supporting the involvement of sirtuins in regulating this stress response [41]. The presence of foamy macrophages in NR8383 cells following CNT exposure has previously been reported Fujita et al., who additionally observed granuloma formation in vivo after exposing rats to the same MWCNT [14]. These results suggest a link between foam cell formation and granuloma development. Similar disruptions in lipid homeostasis were previously observed in this cell model following exposure to metallic oxide nanoparticles, indicating that such responses may reflect a broader “nano” effect on macrophage lipid metabolism [23].

Imaging of NR8383 ells following exposure to MWCNTs (specifically Mitsui-7) revealed the formation of syncytia—multinucleated giant cells resulting from the fusion of monocytes and macrophages, typically observed in response to infection or chronic inflammation [42]. Overexpression of the Cd14 gene receptor, a marker commonly associated with giant cell formation, was also detected [43]. Syncytium formation is often promoted by cytokine secretion [42,44,45,46,47], and Mitsui-7 exposure has been shown to induce the expression of several inflammatory genes, including Ccl2, Ccl6, Nfil3, Ciapin1, Il-1b, and Ilf3. In this current study, inflammatory proteins such as Il7, Il1a, and Il7r. were also identified in the WCL og NR8383 cells after 4 h MWCNT exposure. These findings suggest that Mitsui-7 MWCNTs promote macrophage and monocytes fusion by inducing an inflammatory reaction, resulting in the formation of multinucleated giant cells (Figure S5). The presence of both syncytia and foamy macrophages—hallmarks of granuloma formation—further supports this conclusion [42,47,48,49,50].

Previous in vivo studies have demonstrated that Mitsui-7 induces granuloma formation [8]. The current *in vitro* data validates these observations and identify key cellular events that may precede granuloma development. As shown in Figure 3, hormesis was observed after 4h exposure to MWCNT [51]. Moreover, based on the detection of Il-1a in WCL of these cells, it can be concluded that MWCNT, by inducing Il1a secretion, promote macrophages proliferation and differentiation, thereby endowing them with critical phagocytic functions and organizing granuloma formation, as previously demonstrated by Huaux et al. [52].

Similar responses are observed in dTHP-1 cells following exposure to MWCNT. Overexpression of mitochondrial ribosomal proteins, deregulation of Sirtuin signaling, endoplasmic reticulum dysfunction, and perturbations in lipid metabolism were detected. In contrast to alveolar macrophages, no formation of foamy macrophages or syncytia were observed. Instead, DEPs in dTHP-1 cells were associated regulated exocytosis and immune responses.

The results highlight the critical role of the protein corona formed on carbon nanotubes (CNTs) in modulating cellular responses. Both multi-walled CNTs (MWCNTs) and single-walled CNTs (SWCNTs), when dispersed in fetal bovine serum (FBS) or low serum (LS) media, acquired distinct protein coronas that influenced their interaction with macrophages. Notably, differentially expressed proteins (DEPs) related to purine ribonucleotide binding were identified when CNTs were dispersed in FBS, whereas DEPs associated with syntaxin and SNARE binding, as well as response to toxic substances, were observed with MWCNTs dispersed in HS (low serum). Since Syntaxin, SNARE, and purine signaling pathways are implicated in macrophage polarization, these findings suggest that differentiated dTHP-1 cells become activated and may initiate an effective immune response upon MWCNT exposure, though through different signaling pathways depending on the MWCNT corona composition [43,44,45,46,47,48,49,50,51,52,53,54,55,56]. Furthermore, lysosomal exocytosis may be triggered by the overexpression of TFEB (FC = 6) and SNARE complex formation in dTHP-1 cells exposed to MWCNT dispersed in HS, contributing to lipid homeostasis and the prevention of foamy macrophage formation [57]. The absence of foamy macrophages in dTHP-1 cells may also be attributed to their differentiation state, as concluded by Most et al. [42]. These pathways—well-known regulators of vesicle-mediated transport and exocytosis—indicate that protein corona composition modulates intracellular trafficking and secretion processes, thereby influencing macrophage polarization, immune activation, and the cellular fate of CNT exposure. This is consistent with previous studies demonstrating that the protein corona actively governs nanoparticle recognition, uptake, and downstream signaling responses [58,59]. Corona-mediated modulation of immune and lipid metabolism pathways underscores its pivotal role in determining CNT toxicity, granuloma formation, or cytotoxicity, depending on CNT type and dispersion conditions.

In NR8383 cells, SWCNTs, similar to MWCNTs, induced deregulation of ribosomal proteins, as well as perturbations in the mTOR and eif2 signaling pathways. Complement proteins contributing to the inflammatory response were also detected in the supernatant of NR8383 cells exposed to both types of CNTs [60].

However, SWCNTs—unlike MWCNTs—induced dose-dependent cell death, along with splicing and RNA metabolism disruption in NR8383 cells. Similar observations were reported by Ndika et al., who identified altered spliceosome and ribosomal activities in MRC9, A549, and human macrophages following SWCNT exposure [61]. According To Ndika et al., dysfunction ofmultiprotein complexes such as endoplasmic reticulum and the spliceosome is associated with CNT-induced cytoskeleton damage. These findings are consistent with the presented results, particularly given the deregulation of certain cytoskeleton-related genes such as Ckap5, Ckap2, Tuba1c, Tuba3a, Tpgs2, Myl4, Myo5a and the proteins Map1lc3b, Tbce, Myo5a, and Rhot2. Disruption of the spliceosome may be attributed to the high metal impurity content of SWCNTs, as spliceosome activity is known to depend on catalytic metal ions [62]. In contrast, this effect was not observed with MWCNTs, which contain fewer metal impurities, potentially explaining the differential responses induced by the two CNT types. The significant macrophage cell death observed after SWCNT exposure may result from the combined impairment of ribosomal and spliceosomal functions—both of which are essential for maintaining cellular homeostasis. In comparison, MWCNTs appear to affect only ribosomal activity, without a comparable impact on cellular viability. These findings corroborate those of Ndika et al. and further [61] support the potential utility of ribosomal and spliceosomal alterations as diagnostic markers of CNT exposure. Additionally, SWCNTs enhanced NR8383 cell activation; as evidenced by the overexpression of Tac4 [63,64], Gdf15 [65], Cxcl2 [66], Phf19 [67], and Osm genes [68]. The detection of serpin proteinsc in the cell supernatant further supports macrophage activation in response to SWCNT exposure [69].

SWCNTs did not elicit the same effects in dTHP-1 cells as observed in NR8383 cells, neither cytotoxicity nor spliceosome dysfunction was detected Instead, transcriptomic data indicated deregulation of genes associated with translation. However, this effect was not reflected at the protein level after 24h, with only limited overlap between differentially expressed genes and corresponding proteins—similar to observations with MWCNT. These findings suggest that dTHP-1 cells may possess mechanisms to regulate protein homeostasis following CNT exposure.

## 5. Conclusions

Analysis of dysregulated pathways into two distinct cell models highlights the importance of CNT length in determining toxicity. Nevertheless, differences in cellular responses were also observed, attributable to variations in the physical and chemical characteristics of the studied CNTs, particularly in diameter and metal impurity content. In the case of MWCNTs, perturbations in lipid metabolism dependent on endoplasmic reticulum function were detected in both cell lines; however, lipid accumulation occurred only in NR8383 cells. This suggests that the regulated exocytosis observed in dTHP-1 cells may prevent the formation of foam cells. In contrast, lipid accumulation in NR8383 cells was accompanied by syncytia formation—two key events associated with granuloma development—previously reported in vivo following exposure to this MWCNT. 

For SWCNTs, the higher metal impurity content was associated with spliceosome disruption and subsequent cytotoxicity in NR8383 cells, whereas no toxic effects were observed in dTHP-1 cells. Overall, the findings indicate that SWCNTs with smaller diameters tend to induce cytotoxicity, while MWCNTs with larger diameters promote cell proliferation and exhibit the potential to induce granuloma formation. Furthermore, NR8383 alveolar macrophages appear to be more affected by CNT exposure than differentiated dTHP-1 macrophages.

## Figures and Tables

**Table 1:** Physicochemical Characterization of CNTs by Electron Microscopy and DLS.

Property		NRCWE-006 MWCNT	NRCWE-055 SWCNT
**Length (µm)**		3 ± 2	2 ± 1
**Diameter (nm)**		65 ± 3	12 ± 2
**Metal Impurities (%)**	Fe_2_O_3_	1.11	60.74
	CoO	0	19.27
	Al_2_O_3_	0	0.14
	MgO	0.18	0.42
	Other	0.0068	19.43
**Slope fluorescein/(cm^2^/mL)**		1302	5571
**Surface Area/BET (m^2^/g)**		26	453
**Hydrodynamic Diameter (nm) in FBS**		557 ± 63	601 ± 90
**Hydrodynamic Diameter (nm) in HS + HA**		1023 ± 56	1129 ± 74

## Data Availability

Data will be available upon request.

## Supplementary Materials

The following supporting information can be downloaded at https://www.mdpi.com/article/10.3390/nano15181401/s1, Table S1: Top Ten Differentially Expressed Proteins in NR8383 and dTHP-1 Cells After 4-h MWCNT and SWCNT Exposure; Figure S1: Lipid Metabolism-Related Differentially Expressed Proteins (DEPs) Unique to the Proteome of Differentiated dTHP-1 Cells After 24-h MWCNT Exposure (Whole Cell Lysate); Figure S2: Common Differentially Expressed Proteins (DEPs) Related to Regulated Exocytosis and Immune Response in Supernatants of Differentiated dTHP-1 Cells After 24 h MWCNT or SWCNT Exposure; Figure S3: Main clusters of common and distinctive DEP between WCL of dTHP-1 exposed to CNT dispersed in FBS and WCL of those exposed to CNT dispersed in HS; Figure S4: Main clusters of common and distinctive DEP between supernatant of dTHP-1 exposed to CNT dispersed in FBS and WCL of those exposed to CNT dispersed in HS; Figure S5: Images of NR8383 cells stained according to MGG technique, after exposure for 4 h and 24 h to MWCNT at 1 cm^2^/cm^2^.

## Abbreviations

The following abbreviations are used in this manuscript:

CNT	carbon nanotubes
DEGs	differentially expressed genes
DEPs	differentially expressed proteins
FBS	fetal bovine serum
HA	human albumin
HS	human surfactant
MWCNT	multi-walled carbon nanotubes
PMA	phorbol 12-myristate 13-acetate
SWCNT	single-walled carbon nanotubes
