# Supplementary material for: Single- vs. Multi-Walled Carbon Nanotubes: Differential Cellular Stress and Lipid Metabolism Effects in Macrophage Models
Source: Nanomaterials (Basel). 2025 Sep 11;15(18):1401. doi: 10.3390/nano15181401 (PMC12472611; doi:10.3390/nano15181401)
Supplement: Supplementary file 1 [file nanomaterials-15-01401-s001.zip › nanomaterials-3703138-supplementary.pdf]

Supplementary material

Table S1. Top Ten Differentially Expressed Proteins in NR8383 and dTHP-1 Cells After 4-Hour MWCNT and SWCNT Exposure.

MWCNT (Mitsui-7)				SWCNT (NRCWE-055)			
NR8383		Thp-1		NR8383		Thp-1	
Emp1	18,79	TMEM119	218.75	Tac4	46,52	VTRNA1-3	196.03
Rgcc	14,66	HSPA1A	172.30	Gdf15	18,81	TMEM119	178.69
Csrnp1	12,94	TNFRSF25	153.97	Cxcl2	17,84	TNFRSF25	148.93
Vom2r-ps135	11,43	VTRNA1-3	114.77	Cpa1	14,93	HSPA1A	146.95
Dusp2	11,32	WNT1	114.11	Phf19	14,66	ARPC5	82.69
S100a6	9,64	ARPC5	95.82	Osm	13,95	PTGDS	77.87
Cdc42ep2	9,13	PTGDS	84.53	Sohlh2	10,99	WNT1	75.11
Ccl2	8,96	lnc-AC006156.2-6	67.54	Mafb	10,21	SNORA44	61.75
Hilpda	8,36	MFAP4	64.86	Ascl3	9,79	STATH	61.57
Jund	7,78	CSPG4	64.01	Myl4	9,60	lnc-KIAA0319L-1	60.80

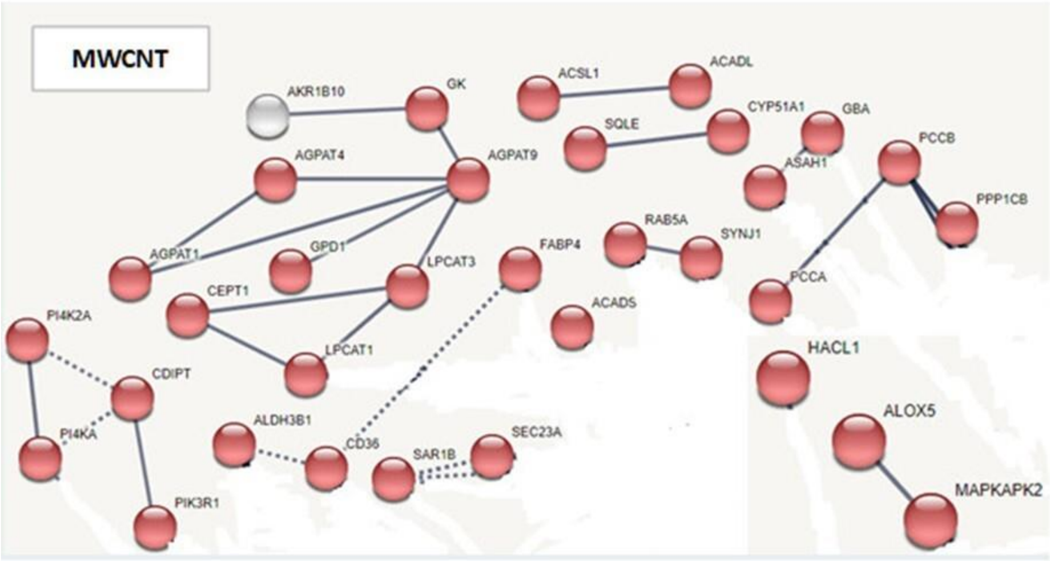

Figure S1: Lipid Metabolism-Related Differentially Expressed Proteins (DEPs) Unique to the Proteome of Differentiated dTHP-1 Cells After 24-Hour MWCNT Exposure (Whole Cell Lysate).

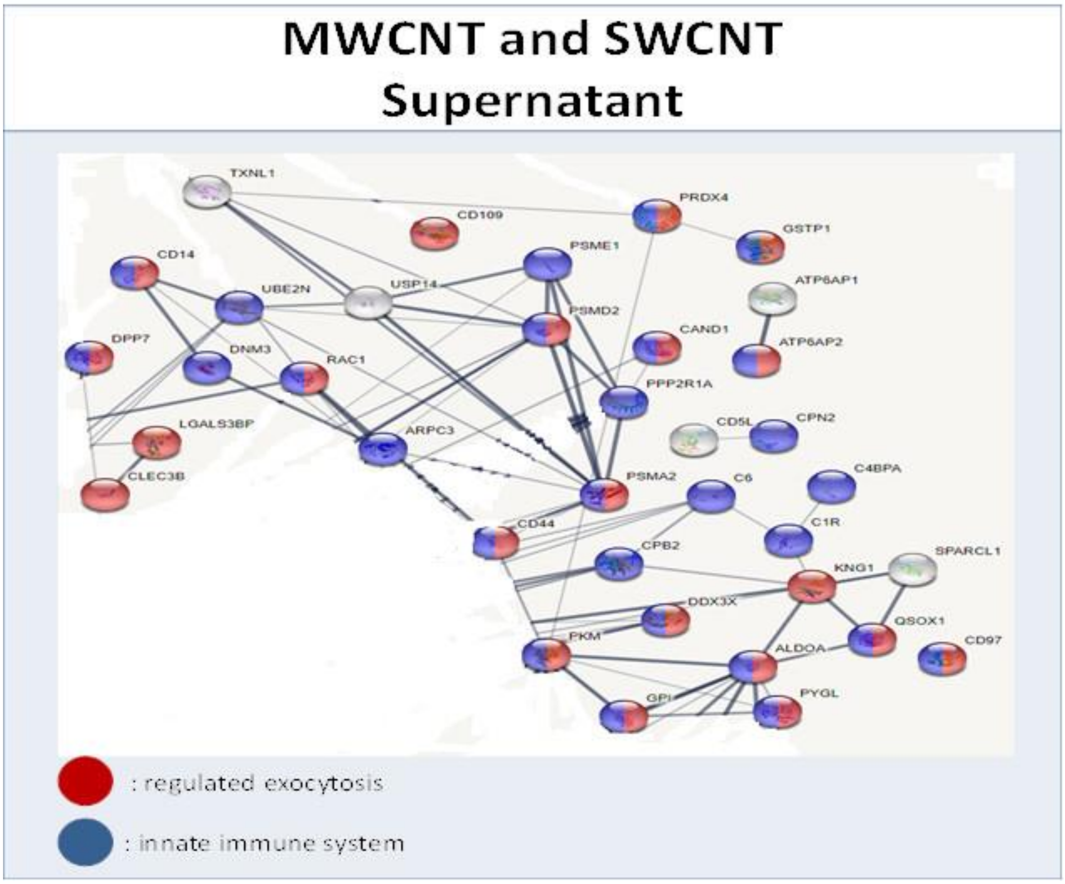

Figure S2. Common Differentially Expressed Proteins (DEPs) Related to Regulated Exocytosis and Immune Response in Supernatants of Differentiated dTHP-1 Cells After 24 Hour MWCNT or SWCNT Exposure.

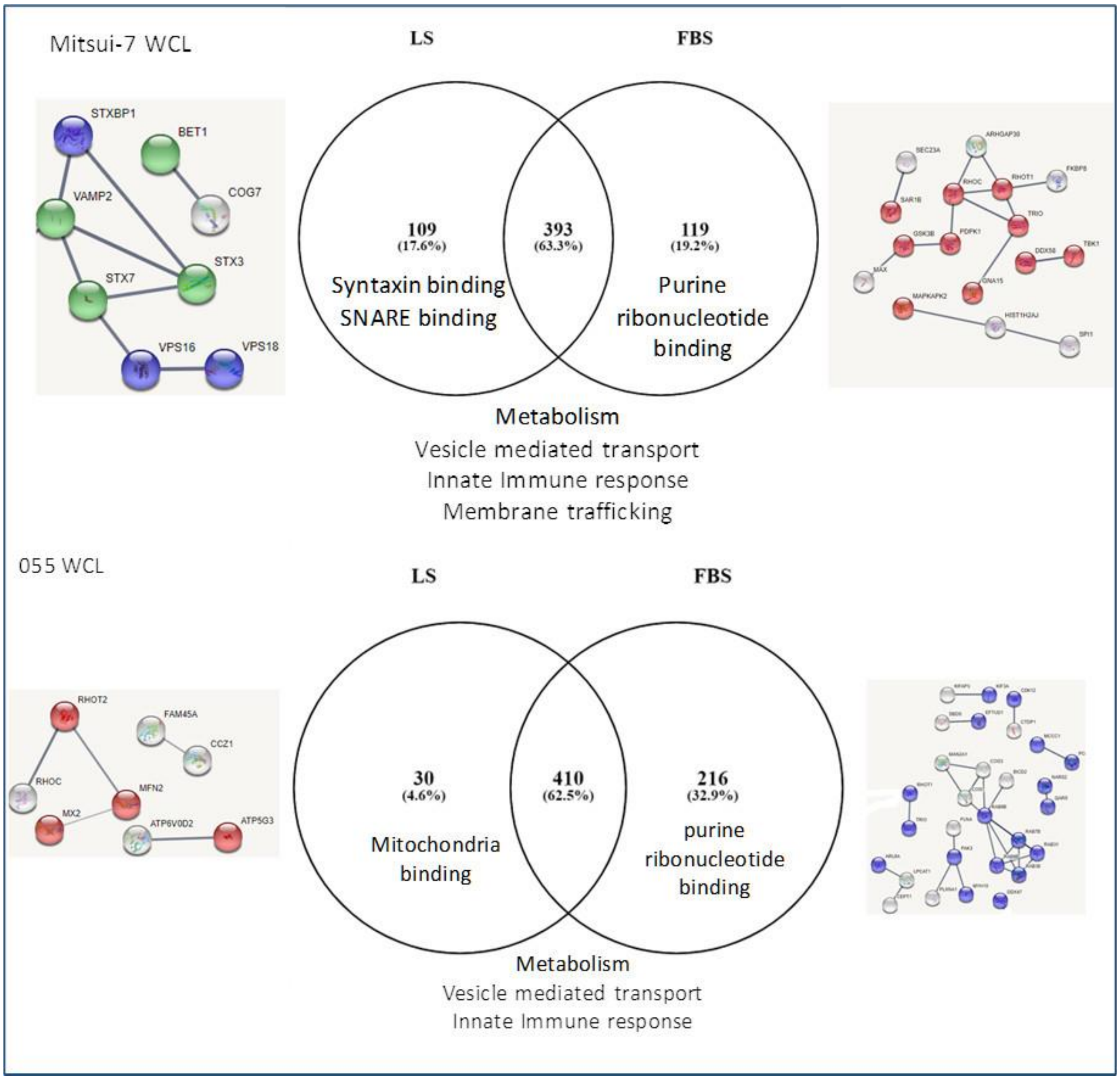

Figure S3. Main clusters of common and distinctive DEP between WCL of dTHP-1 exposed to CNT dispersed in FBS and WCL of those exposed to CNT dispersed in HS.

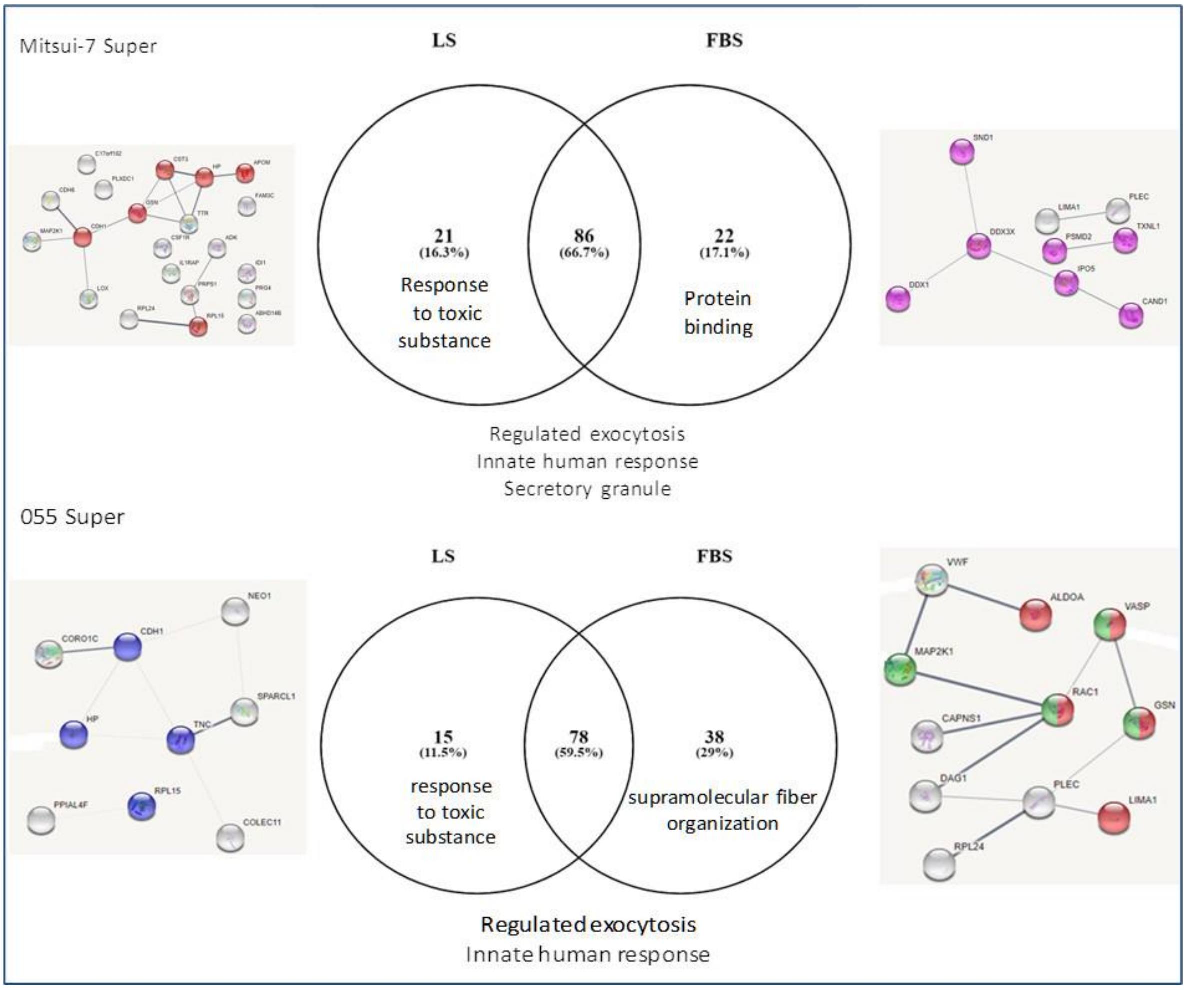

Figure S4. Main clusters of common and distinctive DEP between supernatant of dTHP-1 exposed to CNT dispersed in FBS and WCL of those exposed to CNT dispersed in HS.

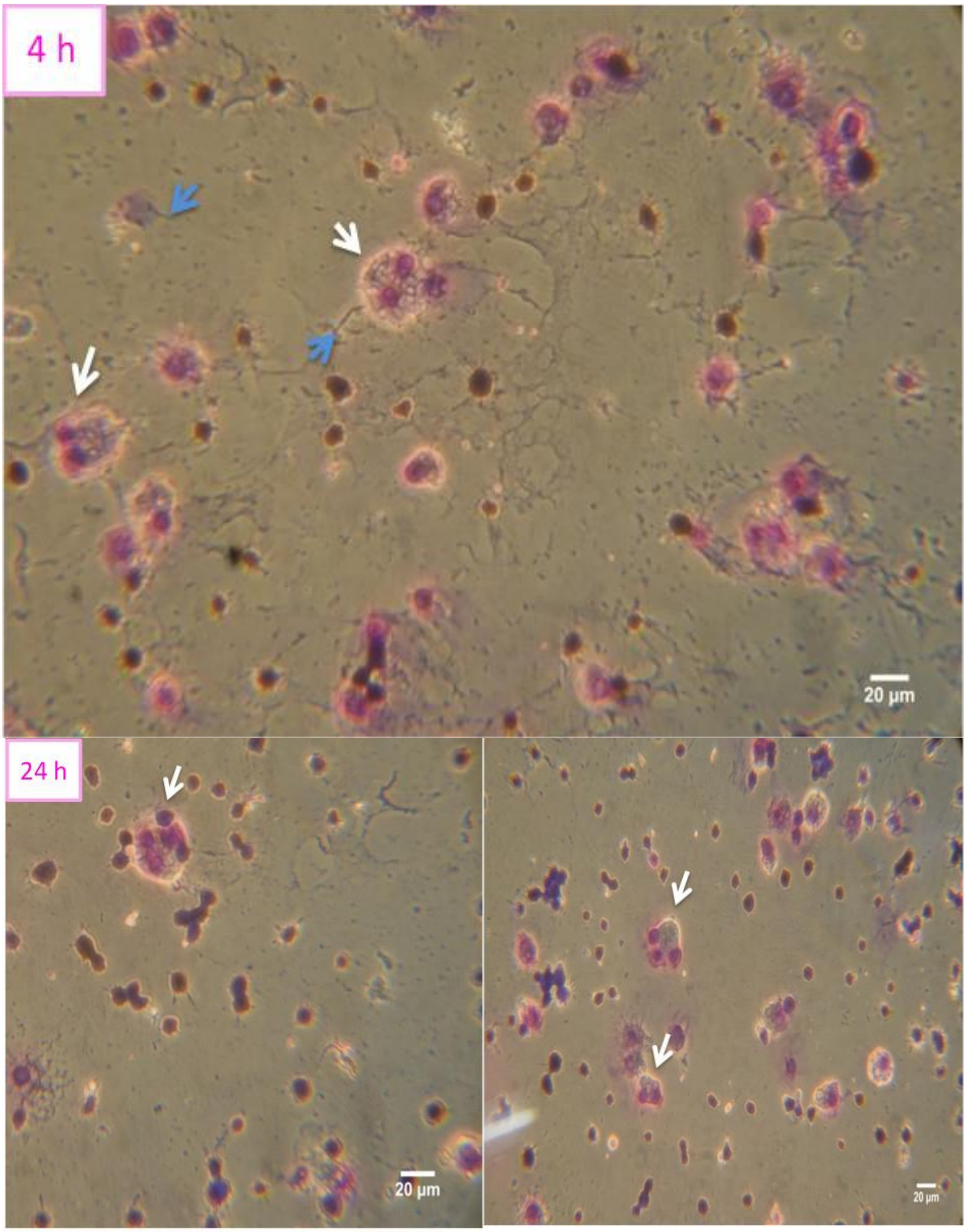

Figure S5. Images of NR8383 cells stained according to MGG technique, after exposure for 4h and 24h to MWCNT at 1 cm^2/cm^2. The presence of syncytia (white arrows) and foamy macrophages following exposure of NR8383 cells to MWCNT for 4 and 24h. MWCNT are designated by blue arrows.
